# Correlations of CSF Biomarkers of Alzheimer’s Disease with Cognitive Measures in MCI and AD Dementia: A Cross-Sectional Analysis

**DOI:** 10.21203/rs.3.rs-8168314/v1

**Published:** 2025-12-01

**Authors:** Gabriel Bitar, Sierra Alban, Kassu Beyene, Boris Decourt, Shervin Harirchian, Marwan N. Sabbagh

**Affiliations:** Creighton University School of Medicine; Creighton University School of Medicine; Barrow Neurological Institute; Texas Tech University Health Sciences Center; Creighton University School of Medicine; Barrow Neurological Institute

**Keywords:** Alzheimer’s Disease Neuroimaging Initiative, Amyloid-β, Mild cognitive impairment, Mini-Mental State Exam, Montreal Cognitive Assessment, Phosphorylated-tau, preclinical disease, Total-tau

## Abstract

**Background and Objectives:**

We examined the relationships between cerebrospinal fluid (CSF) amyloid-β, tau, p-tau, and cognition in an Alzheimer’s Disease Neuroimaging Initiative (ADNI) dataset. Studies have examined the longitudinal relationships between CSF biomarkers for Alzheimer’s disease (AD) and cognition, but there is discordance in the strength of associations between CSF amyloid-β, t-tau, or p-tau with cognition at different disease stages.

**Methods:**

The study included 665 patients from the combined ADNI dataset: 128 cognitively normal (CN), 175 with mild cognitive impairment (MCI), and 362 with AD dementia. All patients were amyloid-β–positive according to established CSF amyloid-β cut-off values. Cognitive assessments, diagnoses, and specimen collection/processing were conducted via published standardized protocols. We examined cross-sectional baseline data and performed correlational and regression analyses to evaluate the associations between CSF biomarkers and assessments of learning, memory, executive function, language, attention, visuospatial skills, and activities of daily living.

**Results:**

In the MCI cohort, significant negative correlations were observed between CSF amyloid-β and ADAS-Cog11 (r=−0.164, p=0.02, ADAS-Cog13 (r=−0.181, p=0.01), Trails B (r=0.11, p=0.04), and FAQ (r=−0.131, p=0.01). There were positive correlations between amyloid-β and MMSE (r=0.149, p=0.004) and amyloid-β and WMS-delayed recall (r=0.116, p=0.03). Statistically significant correlations were observed between CSF t-tau and p-tau and CDRSB (t-tau: r=0.105, p=0.047), ADAS-Cog11 (t-tau: r=0.114, p=0.03; p-tau-181: r=0.114, p=0.03), ADAS-Cog13 (t-tau: r=0.165, p=0.002; p-tau-181: r=0.162, p=0.002), RAVLT-forgetting (t-tau: r=0.173, p=0.001; p-tau-181: r=0.167, p=0.001), and WMS-delayed recall (t-tau: r=−0.237, p<0.001 and p-tau-181: r=−0.235, p<0.001). In the AD cohort, no statistically significant relationships were observed between CSF amyloid-β_1–42_, t-tau, p-tau-181, and any of the cognitive scores.

**Discussion:**

The findings suggest that the relationship between CSF biomarkers and cognitive performance is strongest in MCI. The lack of significant correlations in the AD cohort may indicate other pathophysiological changes dominating cognitive dysfunction at this disease stage. Both tau and amyloid showed similar utility in reflecting cognitive impairment, in contrast to some reports in the literature. Further research is warranted to explore biomarker longitudinal impacts and their predictive values across the spectrum of cognitive impairment.

## BACKGROUND

As our aging population continues to increase, Alzheimer’s disease (AD) prevalence and the associated cost burden rise dramatically.^1^ The hallmarks of AD include amyloid-β (Aβ) plaques and neurofibrillary tangles of tau accumulating in the brain.^2^ Current clinical guidelines use neuroimaging and cognitive testing, such as the Montreal Cognitive Assessment and Mini Mental State Exam (MMSE), among others, to diagnose patients and determine the level of decline.^3,4^ Often, by the time cognitive decline has begun, significant brain changes have already occurred during the “preclinical” phase of the disease.^5,6^ In recent years, researchers have studied the potential of biomarkers to help diagnose AD earlier and initiate interventions as soon as possible, perhaps before clinical manifestations occur.^7^ Despite advances in biomarker discovery it remains unclear how traditional CSF biomarkers relate to cognition at different stages of Alzheimer’s Disease within a well-characterized cohort.

Cerebrospinal fluid (CSF) biomarkers consisting of Aβ_1–42_, total-tau (t-tau), and phosphorylated-tau (p-tau) have been shown to reflect the disease course and may even indicate preclinical disease.^8–11^ Typically, decreased CSF Aβ_1–42_ and increased t-tau and p-tau reflect AD plaque accumulation and neurodegeneration, respectively. While these biomarkers are widely used for research purposes, positron emission tomography (PET), CSF, and blood-based tests are being used more commonly in clinical settings for diagnosis.^12–15^

Previous studies have shown tau, on brain PET and in CSF, to be more closely related to the degree of cognitive decline and likely precedes symptom onset compared to Aβ.^5,16,17^ However, some investigators reported conflicting results, suggesting a minimal relationship between CSF biomarkers and cognitive performance or proposing alternate biomarker methods altogether.^18–20^ Hansson and colleagues proposed that CSF biomarkers agree well with imaging in predicting future decline.^7^ Given the discordant results in the literature, there are gaps in our understanding of the role of CSF Aβ and tau in cognitive decline.

Our study is one of the first to examine the relationships between CSF biomarkers in the Alzheimer’s Disease Neuroimaging Initiative (ADNI) cohort and all available cognitive tests. The primary aim of this study was to elucidate the cross-sectional relationships between CSF biomarkers and cognitive performance across different stages of cognitive impairment. By leveraging an extensive, well-characterized ADNI dataset, we sought to clarify discordances in prior literature regarding biomarker significance and provide insights into their clinical utility. The objective of this study was to systematically examine cross-sectional relationships between CSF Aβ_1–42_, t-tau, and p-tau-181 and multiple domains of cognition across CN, MCI, and AD groups using the ADNI dataset. In accordance with prior literature, we hypothesize that CSF biomarkers, particularly p-tau and t-tau, will demonstrate stronger correlations with cognitive decline measures than Aβ_1–42_, and that these associations will vary across different cognitive domains and stages of impairment.

## METHODS

The study evaluates the associations between CSF biomarker expressions at baseline within various cognitive domains in a large, robust dataset. We hypothesize that CSF biomarkers, particularly p-tau and t-tau, would demonstrate stronger correlations with cognitive decline measures than Aβ_1–42_, particularly in the cohort with mild cognitive impairment (MCI) and that these associations may vary across the spectrum of impairment.

### Subjects and Variables

Study data were obtained from the combined ADNI 1, 2, and GO database (adni.loni.usc.edu). The ADNI was launched in 2003 as a public-private partnership led by Principal Investigator Michael W. Weiner, MD. The primary goal of the ADNI has been to assess whether serial magnetic resonance imaging (MRI), (PET, other biological markers, and clinical and neuropsychological assessment can be combined to measure the progression of mild cognitive impairment (MCI) and early AD.

The study cohorts consisted of people who were classified as cognitively normal (CN), having MCI, or having AD on the basis of standardized diagnostic criteria. For inclusion in the study, participants had to be 55–90 years of age and have a clinical diagnosis of AD, MCI, or CN, with no history of other neurological disorders. Exclusion criteria included conditions that could confound the results, such as major psychiatric disorders or significant systemic illnesses as defined by the ADNI protocol. We additionally excluded those with a history of stroke or a cerebrovascular event. MCI was diagnosed on the basis of evidence of concern for cognitive changes and diminished performance in 1 or more cognitive domains but with generally intact functional ability and lack of significant impairment of social or occupational functioning.^21^ AD was diagnosed as cognitive challenges interfering with daily activities, a decline from previous functioning, and cognitive impairments detected on cognitive testing.^22^ Participants with normal cognition did not exhibit significant cognitive changes that aligned with MCI or AD diagnoses.

Data were retrieved in August 2024 from ADNIMERGE, a curated database that consolidates key clinical, biomarker, and imaging data from the ADNI dataset. The dataset included CSF biomarker levels, cognitive assessments, and demographic variables. A total of 1017 patients were initially queried, with 976 remaining after selection for those with available CSF biomarker data. These 976 patients were further selected on the basis of their amyloid status to 665, which were considered Aβ+, as amyloid positivity is a key biomarker criterion for defining Alzheimer’s disease-specific cohorts. The Aβ + cut-off was determined by methods established and validated, using a cut-off of ≤ 980 pg/mL.^23^ The cut-off was determined on the basis of concordance with amyloid PET data in the BioFINDER cohort and then validated in an independent ADNI cohort.^7^ Demographic variables such as age and education were matched across diagnostic groups (CN, MCI, AD).

CSF data were acquired from the ADNIMERGE. The data were analyzed using the xMAP Luminex immunoassay platform, which was published and validated by the University of Pennsylvania Biomarker Core. Detailed descriptions of the assay and its validation can be found in the literature and ADNI protocol documents.^11^

Cognitive function was assessed using 7 standardized instruments including: (1) the Clinical Dementia Rating Scale Sum of Boxes (CDRSB), which measures global cognitive and functional impairment; (2) the Alzheimer’s Disease Assessment Scale-Cognitive Subscale (ADAS-Cog 11, ADAS-Cog 13), which evaluates cognitive performance, including memory, language, and praxis; (3) the MMSE, which screens for global cognitive function; (4) the Rey Auditory Verbal Learning Test (RAVLT), which assesses verbal memory and learning; (5) the Trail Making Test Part B (Trails B), which evaluates executive function and cognitive flexibility; (6) the Logical Memory Delayed Recall (LDELTOTAL), which measures delayed episodic memory recall; and (7) the Functional Activities Questionnaire (FAQ), which assesses instrumental activities of daily living.

### Statistical Analysis

Descriptive summary statistics were calculated, including means and standard deviations for continuous variables and frequencies and percentages for categorical variables. To explore the distribution of both CSF biomarkers and cognitive measures across cognitive status (i.e., CN, MCI, and AD), the violin plots were generated. The relationship between CSF biomarkers, cognitive measures or continuous demographic variables and cognitive status were assessed using one-way analysis of variance (ANOVA), and post hoc pairwise comparisons between cognitive statuses performed using t-test with Bonferroni correction to control for multiple testing. The association between the categorical demographic variable sex and cognitive status was assessed using the Chi-squared tests. The results were presented as frequencies and percentages, with p-value indicating the significance of association. The relationships between continuous variables CSF biomarkers and cognitive measures were visualized using scatter plots (Supplemental Fig. 1) and quantified using Pearson correlation analysis. Multivariable linear regression model was employed to explore the association of CSF biomarkers with cognitive assessments, adjusting for potential confounders such as age, sex, and education. All statistical analyses were performed using R statistical software (version 4.4.1, R Foundation for Statistical Computing, Vienna, Austria), with a 2-sided significance level of 5% for hypothesis tests.

### Standard Protocol Approvals, Registrations, and Patient Consents

Data used in the preparation of this article were obtained from the Alzheimer’s Disease Neuroimaging Initiative (ADNI) database (Adni.loni.usc.edu). The ADNI study was approved by the institutional review boards (IRBs) of all participating institutions, and written informed consent was obtained from all participants or their authorized representatives. This retrospective project used only deidentified, publicly available data. Therefore, our analysis was exempt from additional IRB review due to the extremely low likelihood of patient identification.

### Data Availability

The data that support the study findings are available from the corresponding author upon reasonable request and IRB approval, as applicable.

## RESULTS

A total of 665 individuals from the combined ADNI 1, 2, and GO database were included in this analysis, categorized into 3 groups: CN (n = 128), MCI (n = 175), and AD (n = 362). Pearson correlations were performed to examine the relationship between CSF biomarkers (Aβ_1–42_, t-tau, p-tau-181) and various cognitive assessment scores across the groups.

### Summary Statistics and Associations

Significant differences (p < 0.05) were observed for all variables analyzed (Table 1), including CSF biomarkers Aβ_1–42_, t-tau, and p-tau-181, demographic factors, and cognitive measures CDRSB, ADAS-Cog11, ADAS-Cog13, MMSE, RAVLT Immediate, WMS-delayed recall, Trails B, and FAQ. These differences highlight the distinct profiles of cognitive and biomarker characteristics across the spectrum of diagnostic stages.

These group differences are further illustrated in Fig. 2, which displays the distribution of CSF biomarkers and cognitive scores across CN, MCI, and AD groups, along with ANOVA and post-hoc comparisons.

### Regression Analysis

Multiple linear regression analysis, adjusted for demographic variables (age, sex, education) (Table 2), confirmed that CSF Aβ_1–42_ was significantly negatively associated with cognitive decline measures such as CDRSB (estimate=−0.002, p < 0.001), ADAS-Cog11 (Estimate=−0.009, p < 0.001), and ADAS-Cog13 (estimate=−0.014, p < 0.001). Similar trends were observed for t-tau and p-tau-181, which were positively associated with markers of cognitive impairment (e.g., CDRSB, ADAS-Cog13) and negatively associated with measures of preserved function (e.g., MMSE, RAVLT-immediate, WMS-delayed recall).

### Correlation Analysis

#### MCI Cohort

In the MCI group, significant correlations were observed between CSF biomarkers and cognitive assessments. Negative correlations were noted between CSF Aβ_1–42_ and the following cognitive measures: ADAS-Cog11 (r=−0.164, p = 0.002), ADAS-Cog13 (r=−0.181, p = 0.001), Trails B (r=−0.110, p = 0.04), and FAQ (r=−0.131, p = 0.01). Conversely, positive correlations were identified between CSF Aβ_1–42_ and assessments of memory and global cognitive function, including MMSE (r = 0.149, p = 0.004) and WMS-delayed recall (r = 0.116, p = 0.03).

**Figure 1:**
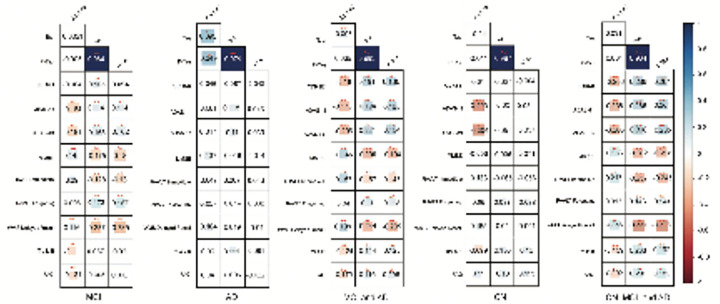
Pearson correlation matrices by cohort. Red asterisks indicate statistical significance (p<0.05). *Used with permission from Barrow Neurological Institute, Phoenix, Arizona.*

**Figure 2:**
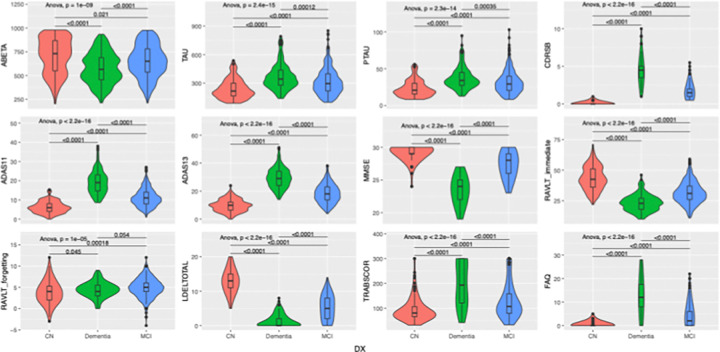
Group differences in CSF biomarkers and cognitive measures across diagnostic categories (CN, MCI, AD)

For CSF t-tau and p-tau-181, significant positive correlations were found with several cognitive measures indicative of cognitive decline (Fig. 1): CDRSB (t-tau: r = 0.105, p = 0.047, ADAS-Cog11 (t-tau: r = 0.114, p = 0.03; p-tau-181: r = 0.114, p = 0.03), ADAS-Cog13 (t-tau: r = 0.165, p = 0.002; p-tau-181: r = 0.162, p = 0.002), RAVLT Forgetting (t-tau: r = 0.173, p = 0.001; ptau-181: r = 0.167, p = 0.001). Negative correlations were observed between t-tau/p-tau-181 and cognitive scores reflecting preserved function, including MMSE (t-tau: r=−0.175, p = 0.001; p-tau181: r=−0.180, p = 0.001), RAVLT-immediate (t-tau: r=−0.128, p = 0.02; p-tau-181: r=−0.130, p = 0.01), and WMS-delayed recall (t-tau: r=−0.237, p < 0.001; p-tau-181: r=−0.235, p < 0.001).

#### AD Cohort

In the AD group, significant correlations were sparse. No statistically significant relationships were observed between CSF Aβ_1–42_, t-tau, p-tau-181, and any of the cognitive assessment scores (Fig. 1).

#### Combined MCI and AD Cohort

When the MCI and AD cohorts were grouped, significant correlations were observed between CSF biomarkers and cognitive measures. CSF Aβ_1–42_ demonstrated significant negative correlations with measures of cognitive decline, including CDRSB (r=−0.160, p < 0.001), ADASCog11 (r=−0.178, p < 0.001), ADAS-Cog13 (r=−0.206, p < 0.001), Trails B (r=−0.124, p = 0.004), and FAQ (r=−0.175, p < 0.001). Positive correlations were noted with global and memory-specific cognitive scores such as MMSE (r = 0.245, p < 0.001), RAVLT Immediate (r = 0.157, p < 0.001), and WMS-delayed recall (r = 0.199, p < 0.001).

CSF t-tau and p-tau-181 exhibited significant correlations with all cognitive measures examined. Both biomarkers were positively correlated with measures of cognitive decline, including CDRSB (t-tau: r = 0.191, p < 0.001; p-tau: r = 0.165, p < 0.001), ADAS-Cog11 (t-tau: r = 0.194, p < 0.001; p-tau-181: r = 0.165, p < 0.001), ADAS-Cog13 (t-tau: r = 0.221, p < 0.001; p-tau181: r = 0.194, p < 0.001), RAVLT Forgetting (t-tau: r = 0.110, p = 0.01; p-tau-181: r = 0.113, p = 0.009), Trails B (t-tau: r = 0.144, p = 0.001; p-tau-181: r = 0.125, p = 0.004), and FAQ (t-tau: r = 0.116, p = 0.007; p-tau-181: r = 0.098, p = 0.02).

Conversely, t-tau and p-tau-181 demonstrated negative correlations with scores indicating better cognitive function (Fig. 1), including MMSE (t-tau: r=−0.209, p < 0.001; p-tau-181: r = 0.198, p < 0.001), RAVLT Immediate (t-tau: r=−0.157, p < 0.001; p-tau-181: r=−0.145, p = 0.001), and WMS-delayed recall (t-tau: r=−0.244, p < 0.001; p-tau-181: r=−0.235, p < 0.001

#### CN Cohort

Significant but modest correlations were found between CSF biomarkers and cognitive measures in the CN group (Fig. 1). CSF Aβ_1–42_ demonstrated negative correlations with ADAS-Cog11 (r=−0.293, p = 0.001) and ADAS-Cog13 (r=−0.304, p < 0.001).

#### Combined CN/MCI/AD Cohort

The strongest relationships between biomarkers and cognitive tests were observed in this cohort, in which all 3 diagnostic groups were combined. CSF Aβ_1–42_ demonstrated significant negative correlations with measures of cognitive decline, including CDRSB (r=−0.206, p < 0.001), ADAS-Cog11 (r=−0.238, p < 0.001), ADAS-Cog13 (r=−0.266, p < 0.001), Trails B (r=−0.159, p < 0.001), and FAQ (r=−0.202, p < 0.001). Positive correlations were noted with global and memory-specific cognitive scores such as MMSE (r = 0.256, p < 0.001), RAVLT Immediate (r = 0.217, p < 0.001), and WMS-delayed recall (r = 0.253, p < 0.001).

CSF t-tau and p-tau-181 again exhibited significant correlations with all cognitive measures examined. Both biomarkers were positively correlated with measures of cognitive decline, including CDRSB (t-tau: r = 0.286, p < 0.001; p-tau: r = 0.264, p < 0.001), ADAS-Cog11 (t-tau: r = 0.276, p < 0.001; p-tau-181: r = 0.251, p < 0.001), ADAS-Cog13 (t-tau: r = 0.308, p < 0.001; ptau-181: r = 0.285, p < 0.001), RAVLT-forgetting (t-tau: r = 0.143, p < 0.001; p-tau-181: r = 0.144, p < 0.05), Trails B (t-tau: r = 0.208, p < 0.001; p-tau-181: r = 0.192, p < 0.001), and FAQ (t-tau: r = 0.202, p < 0.001; p-tau-181: r = 0.185, p < 0.001).

Conversely, t-tau and p-tau-181 demonstrated negative correlations with scores indicating better cognitive function (Fig. 1), including MMSE (t-tau: r=−0.282, p < 0.001; p-tau-181: r = 0.268, p < 0.001), RAVLT Immediate (t-tau: r=−0.257, p < 0.001; p-tau-181: r=−0.245, p < 0.001), and WMS-delayed recall (t-tau: r=−0.331, p < 0.001; p-tau-181: r=−0.323, p < 0.001).

## DISCUSSION

This study examined the relationships between CSF biomarkers (Aβ_1–42_, t-tau, p-tau-181) and cognitive performance across CN, MCI, and AD groups, as well as in a combined MCI/AD cohort. Our findings offer a comprehensive view of biomarker-cognition relationships across the AD continuum, demonstrating that these associations are most pronounced at the MCI stage, where therapeutic intervention may hold the greatest potential impact. Among the most significant findings, Aβ_1–42_ demonstrated correlations with cognitive measures in the MCI group, including negative associations with ADAS-Cog13, ADAS-Cog11, Trails B, and FAQ, and positive associations with MMSE and WMS-delayed recall. Similarly, t-tau and p-tau-181 showed significant correlations with both markers of cognitive decline (e.g., ADAS-Cog13) and preserved cognitive function (e.g., MMSE and RAVLT-immediate). These findings suggest that biomarker levels, particularly in the MCI stage, are closely linked to cognitive performance and may serve as sensitive indicators of early cognitive decline. Additionally, these results suggest that Aβ_1–42_ showed similar significant associations with cognitive measures as tau and p-tau-18, contradicting previous literature that suggested tau to have stronger relationships with cognition^24, 25^. These findings partially support our initial hypothesis: while p-tau and t-tau demonstrated robust associations with cognitive decline in the MCI group, Aβ_142_ also showed significant correlations, particularly with cognitive measures reflecting preserved function. This finding suggests that each biomarker may capture different, yet complementary, aspects of cognitive impairment, and that their relevance may vary by disease stage and cognitive domain affected.

The combined MCI and AD analysis revealed robust correlations across all cognitive measures and biomarkers, reinforcing the importance of this stage in understanding the progression of AD. These findings further highlight the utility of Aβ_1–42_ as a predictor of preserved cognitive function and t-tau and p-tau-181 as markers of neurodegeneration (Fig. 1).

Interestingly, in the AD cohort, significant correlations were sparse. Neither Aβ_1–42_ nor t-tau or p-tau-181 had any significant correlations. These results suggest that in advanced stages of AD, other pathological processes may play a more dominant role in cognitive decline, overshadowing the influence of traditional biomarkers.

In the CN group, significant correlations were limited to Aβ_1–42_, which was negatively associated with ADAS-Cog13 and ADAS-Cog11. These findings indicate that even in individuals with normal cognition, subtle biomarker-cognition relationships can be detected, potentially offering opportunities for early intervention.

This novel study assesses the relationship between well-known cognitive tests and CSF biomarkers of AD in the ADNI dataset. Our findings contribute to the growing knowledge base of the pathophysiology of AD progression. Previous studies have shown tau biomarkers (on PET and in CSF) or a ratio of Aβ/tau to be more closely related to cognition than Aβ alone.^26, 27^ Vemuri and colleagues also illustrated that CSF biomarkers and cognition were not correlated, supporting the use of alternate imaging markers such as MRI of brain atrophy.^19^ Our results illustrate a strong association between Aβ and cognition, suggesting that amyloid accumulation may play a significant role in cognitive decline.

Although the results of this study are promising, we recognize that it has limitations. One primary limitation is the study’s cross-sectional design due to limited sample size of individuals meeting our inclusion criteria. As a result, we cannot conclude how cognition changes over time relate to CSF biomarkers. Over time, the biomarker profile of a patient may change differentially compared to their performance in various domains of cognition. Since ADNI is a large, multi-site data-collecting initiative, there may have been variations in biospecimen collection and processing protocols from 1 site to the next. With the quantity of data collected and the detailed protocol guidelines provided by ADNI, there might have been errors that could have affected our results.

Regarding the ADNI protocol, another potential limitation of the study is the diagnostic categorization of patients as having CN, MCI, or AD, which an on-site physician performs. Although there are specific diagnostic guidelines for physicians, some subjectivity may be inherent in determining this diagnosis. The use of ADNI data also presents a few inherent limitations in its generalizability to the national population and selection biases of subjects involved. Lastly, it would be beneficial to repeat this analysis using PET data since research has shown imaging markers to be increasingly valuable in AD diagnosis, as well as blood-based biomarkers which are less invasive and less costly.^19,28^

Additionally, our study did not include emerging plasma biomarkers, which are now increasingly validated for diagnostic use. Integrating fluid and imaging markers longitudinally will be essential to establish their combined predictive value. Future research should aim to continue improving our diagnostic accuracy of AD, especially early in the disease course, so adequate measures can be taken to slow progression. It would be beneficial to further research CSF biomarker changes over time and how they relate to the clinical course of AD. Additionally, exploring the role of newer cognitive assessment tools and digital phenotyping may enhance our ability to detect subtle cognitive changes in the preclinical and early MCI stages.

## CONCLUSION

In summary, the findings highlight the nuanced relationships between CSF biomarkers Aβ_1–42_, t-tau, and p-tau-181 and cognitive performance across the spectrum of cognitive impairment. We emphasize the importance of early identification and targeted interventions during the MCI stage, where biomarker-cognition correlations are strongest. Future longitudinal studies should aim to validate these findings and explore the predictive value of the biomarkers in disease progression and therapeutic response.

## Supplementary Material

Supplementary Files

This is a list of supplementary files associated with this preprint. Click to download.

• SUPPLEMENTALMATERIALS.docx

## Figures and Tables

**Table 1:** Summary statistics for the variables and results of associations between cognitive function and demographic and other variables.

Variable*	Cognitive function			F or Chi-square statistic	P-value
	CN	MCI	AD		
Age (years), mean (SD)	74.5 (5.81)	73.4 (7.04)	74.3 (7.77)	1.565	0.210
Education (years), mean (SD)	16.3 (2.61)	16.0 (2.82)	15.5 (2.82)	3.596	0.03
CSF Aβ_1–42_ (pg/mL), mean (SD)	703.7 (188.2)	652.1 (170.3)	577.3 (161.4)	21.34	< 0.001
Total tau (pg/mL), mean (SD)	239.6 (100.5)	315.3 (137.4)	367.1 (134.3)	35.42	< 0.001
P-tau-181 (pg/mL), mean (SD)	23.39 (11.21)	31.71 (15.41)	37.05 (14.62)	32.95	< 0.001
CDRSB	0.059 (0.173)	1.627 (0.971)	4.377 (1.624)	626.5	< 0.001
ADAS-Cog11	6.276 (3.060)	11.22 (4.671)	19.19 (6.063)	286.4	< 0.001
ADAS-Cog13	9.792 (4.431)	18.27 (6.673)	29.49 (7.256)	358.9	< 0.001
MMSE	29.10 (1.169)	27.39 (1.886)	23.42 (1.928)	438.7	< 0.001
RAVLT-immediate	43.56 (9.826)	31.51 (8.989)	23.21 (6.726)	205.8	< 0.001
RAVLT-forgetting	3.797 (2.730)	4.898 (2.240)	4.474 (1.774)	11.71	< 0.001
WMS-delayed recall	12.92 (3.264)	5.243 (3.458)	1.331 (1.773)	537.3	< 0.001
Trails B	95.08 (48.27)	129.3 (70.02)	198.7 (86.83)	88.30	< 0.001
FAQ	0.344 (0.846)	3.754 (4.273)	12.79 (6.614)	320.0	< 0.001
Male, n (%)	59 (15.2)	99 (25.5)	230 (59.3)	12.15	0.002
Female, n (%)	69 (24.9)	76 (27.4)	132 (47.6)		

* Data are scores unless otherwise noted.

**Abbreviations:** Aβ, amyloid-β; AD, Alzheimer's disease; ADAS-COG, Alzheimer's Disease Assessment Scale-Cognitive Subscale; CDRSB, Clinical Dementia Rating Scale Sum of Boxes; CN, cognitively normal;CSF, cerebrospinal fluid; FAQ, Functional Activities Questionnaire; MCI, mild cognitive impairment; MMSE, Mini-Mental State Exam; P-Tau, phosphorylated tau; RAVLT, Rey Auditory Verbal Learning Test; Trails B, Trail Making Test Part B.

**Table 2:** Linear regression analysis, adjusted for age, sex, and years of education

Outcomes	Predictors		
	ABETA	TAU	PTAU
	Estimate(p-value)	Estimate(p-value)	Estimate(p-value)
**ALL**			
CDRSB	−0.00214(< 0.0001)	0.00395(< 0.0001)	0.03245(< 0.0001)
ADAS11	−0.00836(< 0.0001)	0.01395(< 0.0001)	0.11320(< 0.0001)
ADAS13	−0.01330(< 0.0001)	0.02175(< 0.0001)	0.18007(< 0.0001)
MMSE	0.00372(< 0.0001)	−0.00561(< 0.0001)	−0.04760(< 0.0001)
RAVLT_immediate	0.01140(< 0.0001)	−0.02334(< 0.0001)	−0.19740(< 0.0001)
RAVLT_forgetting	0.00009(0.85924)	0.00229(0.00048)	0.02084(0.00041)
LDELTOTAL	0.00673(< 0.0001)	−0.01180(< 0.0001)	−0.10256(< 0.0001)
TRABSCOR	−0.06912(0.00007)	0.11684(< 0.0001)	0.96313(< 0.0001)
FAQ	−0.00732(< 0.0001)	0.00985(< 0.0001)	0.08056(< 0.0001)
**CN**			
CDRSB	0.00002(0.81161)	−0.00008(0.63617)	−0.00031(0.82998)
ADAS11	−0.00394(0.00284)	−0.00034(0.89390)	−0.00147(0.94857)
ADAS13	−0.00598(0.00159)	0.00057(0.87654)	0.00790(0.80899)
MMSE	−0.00071(0.18174)	0.00078(0.43762)	0.01077(0.23120)
RAVLT_immediate	0.00298(0.47950)	−0.00013(0.98665)	0.00598(0.93310)
RAVLT_forgetting	0.00126(0.33646)	0.00161(0.51785)	0.01474(0.50726)
LDELTOTAL	0.00196(0.17836)	0.00130(0.63855)	0.00845(0.73279)
TRABSCOR	−0.01103(0.62243)	0.04374(0.30035)	0.42602(0.25965)
FAQ	0.00047(0.25457)	0.00127(0.10150)	0.00994(0.15210)
**MCI**			
CDRSB	−0.00038(0.20472)	0.00078(0.04252)	0.00617(0.07079)
ADAS11	−0.00420(0.00355)	0.00434(0.01766)	0.03810(0.01898)
ADAS13	−0.00674(0.00103)	0.00831(0.00143)	0.07228(0.00178)
MMSE	0.00153(0.00747)	−0.00224(0.00195)	−0.02064(0.00132)
RAVLT_immediate	0.00338(0.21659)	−0.01129(0.00106)	−0.09861(0.00128)
RAVLT_forgetting	−0.00014(0.84492)	0.00273(0.00173)	0.02339(0.00251)
LDELTOTAL	0.00251(0.01589)	−0.00489(0.00019)	−0.04321(0.00020)
TRABSCOR	−0.03990(0.05990)	0.02857(0.28929)	0.22766(0.34143)
FAQ	−0.00318(0.01662)	0.00074(0.66173)	0.00534(0.72201)
**Dementia**			
CDRSB	0.00029(0.71747)	0.00070(0.46936)	0.00123(0.89014)
ADAS11	0.00242(0.41298)	0.00656(0.06848)	0.02669(0.41873)
ADAS13	0.00104(0.76882)	0.00675(0.11796)	0.02469(0.53215)
MMSE	0.00170(0.07012)	−0.00057(0.61862)	−0.00041(0.96888)
RAVLT_immediate	0.00053(0.86846)	−0.00323(0.40455)	−0.00610(0.86327)
RAVLT_forgetting	0.00022(0.79881)	−0.00021(0.84391)	0.00142(0.88310)
LDELTOTAL	0.00150(0.08020)	0.00064(0.54699)	0.00822(0.39290)
TRABSCOR	0.02515(0.54891)	0.04295(0.40232)	0.20139(0.66727)
FAQ	0.00063(0.84653)	−0.00065(0.87079)	−0.01742(0.63031)
**Dementia & MCI**			
CDRSB	−0.00176(0.00009)	0.00227(0.00007)	0.01735(0.00070)
ADAS11	−0.00669(0.00003)	0.00921(0.00001)	0.06978(0.00013)
ADAS13	−0.01067(< 0.0001)	0.01374(< 0.0001)	0.10771(0.00001)
MMSE	0.00387(< 0.0001)	−0.00389(< 0.0001)	−0.03283(0.00001)
RAVLT_immediate	0.00740(0.00116)	−0.01331(< 0.0001)	−0.10915(0.00002)
RAVLT_forgetting	0.00034(0.52916)	0.00145(0.03260)	0.01360(0.02519)
LDELTOTAL	0.00441(< 0.0001)	−0.00514(< 0.0001)	−0.04447(0.00001)
TRABSCOR	−0.06251(0.00242)	0.07558(0.00397)	0.58157(0.01349)
FAQ	−0.00707(0.00003)	0.00534(0.01323)	0.04017(0.03768)

Adjusted for Age, Sex, and Education

**Abbreviations:** CDRSB, Clinical Dementia Rating Scale Sum of Boxes; CN, cognitively normal; CSF, cerebrospinal fluid; FAQ, Functional Activities Questionnaire; MCI, mild cognitive impairment; MMSE, Mini-Mental State Exam; P-Tau, phosphorylated tau; RAVLT, Rey Auditory Verbal Learning Test; Trails B, Trail Making Test Part B.

## Data Availability

Data used in the preparation of this article were obtained from the Alzheimer’s Disease Neuroimaging Initiative (ADNI) database (adni.loni.usc.edu). The ADNI data are publicly available to qualified investigators upon registration and compliance with data use policies. Additional data generated or analyzed during the current study are available from the corresponding author upon reasonable request.

## ABBREVIATIONS

Aβ: amyloid-β
AD: Alzheimer’s Disease
ADNI: Alzheimer’s Disease Neuroimaging Initiative
CN: cognitively normal
CSF: cerebrospinal fluid
IRB: institutional review board
MCI: mild cognitive impairment
MMSE: Mini-Mental State Exam
MoCA: Montreal Cognitive Assessment
MRI: magnetic resonance imaging
PET: positron emission tomography
p-tau: phosphorylated tau
t-tau: total tau
WMS: Wechsler Memory Scale
